# Influence and Mechanism of Azodicarbonamide Expansive Agent on the Workability, Mechanical Strength and Plastic Shrinkage of UHPC

**DOI:** 10.3390/ma18204656

**Published:** 2025-10-10

**Authors:** Haowen Zhan, Jing Yang, Haoran Guo, Caiqian Yang, Weigang Lu, Yuan Yao

**Affiliations:** 1 College of Civil Engineering and Transportation, Yangzhou University, Yangzhou 225127, China; 19551657016@163.com; 2 School of Civil Engineering, Southeast University, Nanjing 210096, China; 202221572259@smail.xtu.edu.cn (H.G.); ycqjxx@seu.edu.cn (C.Y.); 3 College of Hydraulic Science and Engineering, Yangzhou University, Yangzhou 225000, China; wglu@yzu.edu.cn; 4 College of Civil Engineering, Xiangtan University, Xiangtan 411105, China; yjcs1027@163.com

**Keywords:** ultra-high-performance concrete, azodicarbonamide, plastic shrinkage, workability, mechanical properties

## Abstract

This study introduces an innovative approach to addressing the plastic shrinkage of ultra-high-performance concrete (UHPC) using an azodicarbonamide (ADC) expansive agent. The influence of ADC on the workability, mechanical properties, and plastic shrinkage of UHPC were systematically investigated. The findings reveal that the addition of ADC generates a substantial number of bubbles within the UHPC slurry, thereby reducing internal frictional resistance and cohesion of the mixture. Consequently, the fluidity and setting time of UHPC were enhanced to varying degrees with increasing ADC content. However, the introduction of these bubbles also reduced the density, leading to a noticeable decline in both compressive and flexural strength, particularly at later stages. Notably, ADC effectively mitigated early shrinkage and increased the vertical expansion rate within the first 24 h. When the ADC dosage ranged from 0.04% to 0.1%, the UHPC remained in an expanded state within 24 h, with a notable difference in expansion rate exceeding 0.02% from 3 to 24 h. Microstructural and pore structure analysis revealed that the ADC generated considerable gas during the mixing process, forming numerous micropores within the UHPC matrix. These dispersed pores contributed to reduced compactness of the UHPC hydrates, resulting in increased pore area, porosity, and average pore diameter.

## 1. Introduction

Traditional concrete is often characterized by low strength, high brittleness, and limited durability, which restricts its ability to meet future demands in construction and infrastructure development [1,2]. As the industry advances, the focus has shifted toward developing concrete materials with higher strength, improved performance, and longer service life, leading to the emergence of ultra-high-performance concrete (UHPC). As an innovative cement-based material, UHPC is considered one of the most promising solutions in civil engineering due to its exceptional mechanical properties and durability [3,4,5,6].

Despite its superior performance compared to traditional concrete, UHPC still exhibits inherent shortcomings, particularly regarding volume deformation resulting from self-shrinkage and drying shrinkage [3,7,8,9]. Such volume deformation can cause cracking, which results in water and gas infiltration, carbonation, steel corrosion, and chemical erosion, ultimately compromising the durability and functionality of UHPC structures [10,11]. Given its high proportion of cementitious materials, substantial content of active mineral admixtures, and low water-to-binder ratio (W/B), UHPC is prone to significant shrinkage during solidification, hardening, and service phases [12,13,14]. Therefore, addressing the full-process shrinkage of UHPC is crucial for ensuring its widespread application in engineering projects [15].

To control and mitigate the shrinkage problem in UHPC, various strategies have been explored [16]. Soliman and Nehdi [17] introduced wollastonite microfibers to partially replace cementitious materials in UHPC, effectively controlling hydration reactions and reducing shrinkage strain, thus enhancing the cracking resistance of UHPC. YOO et al. [18] investigated the impact of shrinkage-reducing admixture (SRA) on UHPC’s autogenous shrinkage, demonstrating that SRA significantly improved crack resistance and restrained shrinkage behaviors. Meng and Khayat [19] substituted river sand with pre-saturated lightweight sand (LWS) to decrease autogenous shrinkage, which led to a reduction in porosity and a deceleration of UHPC shrinkage.

Furthermore, numerous studies have incorporated expansive agents (EAs) to compensate for UHPC shrinkage, proving to be an effective and economical approach [20,21,22]. EAs are typically categorized as calcium oxide, calcium sulfoaluminate, calcium sulfoaluminate calcium oxide, and magnesium oxide agents. These EAs exhibit varying hydration products capable of generating volume expansion, thereby offsetting shrinkage after hardening [23,24,25]. The shrinkage regulation of UHPC should encompass the entire process, not just the reduction during the later hardening stage. Due to its low water-to-binder ratio and high cementitious material content, UHPC experiences more severe early-age shrinkage compared to traditional concrete [26]. Several studies have investigated methods to mitigate early-age shrinkage. Li et al. [27] found that high-activity MgO expansive agents (MEA) effectively alleviate early autogenous shrinkage, while Liu et al. [9] successfully reduced autogenous shrinkage after 3 days by combining a CaO-based expansive agent (CEA) with super absorbent polymer (SAP) and shrinkage-reducing agent (SRA). Xu et al. [28] reported that the CEA significantly optimized the pore structure of UHPC, leading to a notable reduction in autogenous shrinkage. Du et al. [29] reported the application of pre-wet CEA in solving UHPC shrinkage; the results indicated that the autogenous shrinkage of UHPC with 4% pre-wet CEA was reduced by 20% compared to UHPC with 4% CEA. Additionally, Tian et al. [30], Matos et al. [31], Valipour et al. [32] and Feng et al. [33] explored the use of MEA, SAP, equilibrium catalyst (ECat), and pre-saturated lightweight sand to compensate for early shrinkage in UHPC. Although these studies have made significant progress, insufficient research has been conducted on controlling shrinkage during the plastic stage.

Previous studies focused primarily on solving the hardening stage shrinkage of UHPC, and few studies on how to reduce plastic stage shrinkage of UHPC [34,35,36,37]. However, the shrinkage control during the plastic stage has a significant impact on the performance and development of later shrinkage of concrete [38,39]. The shrinkage compensation effect of ordinary EA is mainly reflected in the hardening stage of concrete, and cannot achieve micro expansion in its plastic stage [40]. The plastic expansive agent (PEA) is widely used in cement-based grouting and sealing materials. It releases gas during the plastic stage of the slurry to achieve volume expansion or prevent volume deformation caused by plastic shrinkage [41,42,43,44,45]. Organic foaming agents like azodicarbonamide (ADC) [41] and triethanolamine [46] are the most common PEAs. Among them, the ADC has the advantages of high gas generation, good dispersion, and stable gas bubbles [41,44]. However, ADC is mainly applied in the foaming of plastics, rubber, and foodstuffs, while its application in concrete has just begun. There are few studies reported addressing the plastic shrinkage problem of UHPC using ADC, which is crucial for its performance improvement and engineering applications.

This paper aims to investigate the effect of ADC on UHPC’s performance, focusing on its role in compensating for shrinkage during the plastic stage. The workability, mechanical properties, and early vertical expansion rates of UHPC with varying ADC content were systematically investigated. Additionally, the pore structure parameters and microstructure of ADC-modified UHPC are analyzed using digital microscopy and scanning electron microscopy (SEM). The findings of this study provide valuable insights into shrinkage control strategies and the practical application of UHPC.

## 2. Experimental Program

### 2.1. Raw Materials

The primary cementitious materials were ordinary Portland cement (OPC, Lianyungang, China) with strength grade 52.5, grade S95 mineral powder (MP, Lianyungang, China), grade I fly ash (FA, Lianyungang, China), and silica fume (SF, Xi’an, China). The main chemical components of cementitious materials are shown in Table 1. To enhance the workability of UHPC, a polycarboxylate superplasticizer (SP, Nanjing, China) with a water reduction rate of ≥30% was utilized. Silica sand (Linyi, China) with a fineness modulus ranging from 2.4 to 2.7 served as the aggregate. The main parameters of the copper-coated steel fiber (CSF, Taian, China) are shown in Table 2.

**Figure 1:**
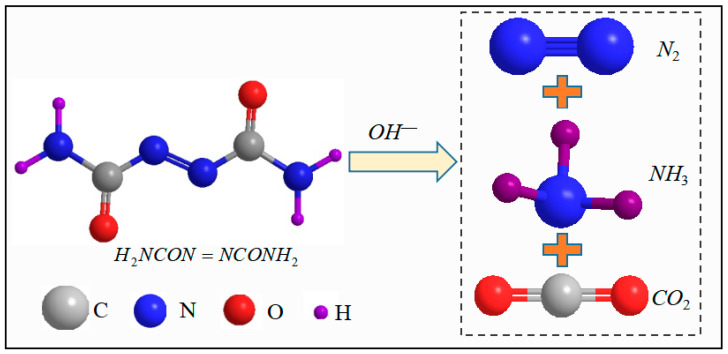
Reaction mechanism of ADC.

ADC (Shanghai, China) was employed as a plastic expansive agent (PEA) to address the plastic shrinkage of UHPC. ADC, with a chemical formula of C_2_H_4_N_4_O_2_, possesses a 24 h expansion coefficient of 0.2%. Notably, the amine nitrogen atoms in ADC lack common electron pairs, and under specific temperatures and the alkaline conditions of cement hydration, the N2 and N3 bonds in ADC break, releasing gases [44]. The chemical reaction is shown in Equation (1) [47,48,49], and the corresponding reaction mechanism is shown in Figure 1. The release of these gases induces volume expansion within the cementitious slurry, resulting in the formation of numerous air bubbles within the concrete matrix. This bubble formation continues until the initial setting time of the concrete [41,48]. The moderate expansion during the plastic stage of the slurry is established, which compensates for volume shrinkage caused by plastic settlement and self-shrinkage of the cement slurry before solidification.

(1) $$\begin{array}{c} H_{2}NCON=NCONH_{2}+2OH^{-}\to {N_{2}C_{2}O_{4}}^{2-}+2NH_{3}↑\\ 2{N_{2}C_{2}O_{4}}^{2-}+2H_{2}O\to N_{2}↑+2CO_{2}↑+N_{2}H_{4}+2C{O_{3}}^{2-} \end{array}$$

### 2.2. Mix Proportion and Specimen Preparation

In this study, an extensive series of experiments was conducted to investigate the effects of plastic expansive agent on the fluidity, setting time, mechanical strength, and shrinkage resistance of UHPC. For optimal construction performance, it is essential that the truncated cone fluidity of UHPC exceeds 240 mm. Additionally, the material should remain free from bleeding or segregation during construction while maintaining adequate fluidity. Based on previous repeated experiments, the dosage of the superplasticizer (SP) was established at 0.45 wt% of the binder content. The sand-to-binder ratio was maintained at 1:1, and the dosage of copper-coated steel fiber (CSF) was fixed at 0.5% by volume. The azodicarbonamide (ADC) dosage varied from 0 to 0.1 wt% of the binder (reference to prior literature [41,49]).

**Figure 2:**
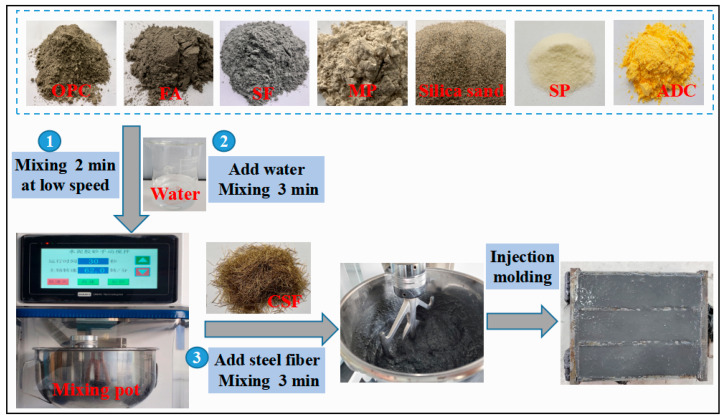
UHPC mixing process employed in this study.

Six different mixtures were designed for this study, including the control group and five experiment groups with varying dosages of ADC. The mixture proportions are listed in Table 3. The UHPC mixing process was divided into the following steps: (1) The cementitious materials, sand, ADC and SP were added to the mixing pot and stirred at a low speed for 2 min; (2) water was then gradually introduced according to the specified water-to-binder (W/B) ratio and mixed at high speed for 3 min; (3) the steel fibers were added slowly and mixed for an additional 3 min until a uniform distribution was achieved. The detailed mixing process is shown in Figure 2.

### 2.3. Testing Method

#### 2.3.1. Workability Tests

**Figure 3:**
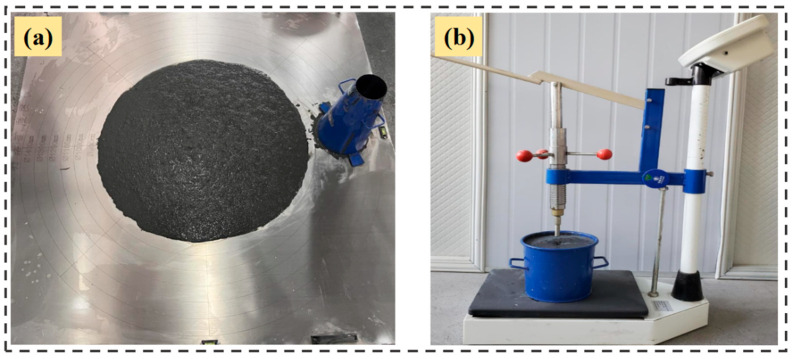
Workability tests: (**a**) fluidity; (**b**) setting time.

The fluidity test of the fresh mixtures was measured in accordance with Chinese standard GB/T 50448-2015 [50]. Each fresh mixture was poured into a truncated circular mold with a height of 60 mm, a top diameter of 70 mm, and a bottom diameter of 100 mm. After the mold was lifted vertically, the diameter of the mixture was measured after 30 s to determine the initial fluidity. Following a 30 min interval, the mixture was stirred for 1 min, and the fluidity at 30 min was assessed using the same method. The initial and final setting times of the mixtures were tested by the penetration resistance method according to Chinese standard GB/T 50080-2016 [51]. The pressure bearing areas of the measuring needle were 100 mm^2^, 50 mm^2^ and 20 mm^2^. According to the condensation condition of the mixture, the measuring needles were replaced by the descending order according to the pressure area during the testing process. The steel needle was gradually inserted to a depth of 25 mm over 10 s. The initial and final setting times were recorded as the times corresponding to penetration resistances of 3 MPa and 28 MPa, respectively. It is important to note that all tests were conducted at a controlled temperature of 20 ± 2 °C. The fluidity and setting time test of the mixtures is shown in Figure 3.

#### 2.3.2. Mechanical Properties

The mechanical properties tests were conducted in accordance with GB/T 17671-2021 [52]. Specimens for both compressive and flexural strength tests were prepared with dimensions of 40 mm × 40 mm × 160 mm. The fresh mixtures were poured into the mold without any vibration. The specimens were initially cured at 20 ± 2 °C and a relative humidity (RH) of 60 ± 5% for 24 h. After this initial curing period, the specimens were demolded and further cured at 20 ± 2 °C with an RH of 95 ± 5% until the designated testing age. Mechanical property evaluations were performed at 1, 3, 7, and 28 days after casting.

#### 2.3.3. Vertical Expansion Rate

**Figure 4:**
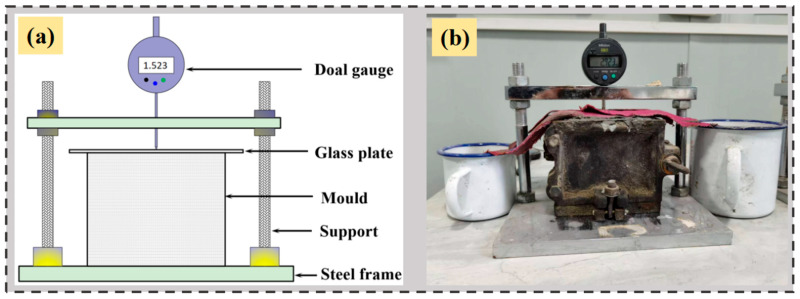
The vertical expansion rate test: (**a**) experiment schematic, (**b**) actual scene.

The vertical expansion rate was tested according to the Chinese standard GB/T 50448-2015 [50]. As shown in Figure 4, the fresh mixtures were poured into molds with the dimensions of 100 mm × 100 mm × 100 mm, with the mixture extending 2 mm above the upper edge of the mold. A glass plate measuring 140 mm × 80 mm × 5 mm was then placed at the center of the upper surface of the mold, ensuring that the mixture adhered closely to the glass plate. The measuring head of the dial indicator was positioned vertically at the center of the glass plate, ensuring that the gauge rod could move freely up and down. Fix the device firmly, and the initial reading *h*_0_ was recorded from the dial indicator within 30 s. The curing conditions were maintained at a temperature of 20 ± 2 °C and a relative humidity (RH) of 60 ± 5%. The readings from the dial indicator were recorded over time as *h*_t_, and the vertical expansion rate *ε*_t_ was calculated according to Equation (2).

(2) $$\varepsilon_{t}=\frac{h_{t}-h_{0}}{h}•100\%$$

where *h* is the height of the specimen, 100 mm.

#### 2.3.4. Pore Structure Parameters

To investigate the effect of ADC on the internal pore structure of UHPC, image analysis methods are adopted for research: (1) Specimens were formed and reached the curing age, after which cut the cross-section as flat as possible, and the cutting thickness controlled at around 2 cm. (2) The cutting surface was polished with an abrasive (fineness of 2000 mesh) for 15–20 min to make the surface smooth and flat. (3) Ink was then applied to the cut surfaces, and Nano CaCO_3_ powder was used to fill the pores. Excess powder was carefully removed from the surface to create an observation area with high black-and-white contrast. (4) A digital microscope (Oplenic, Hangzhou, China) was employed to capture the cross-sectional images of the samples, and the magnification was 100 times, and the Image-Pro Plus software was utilized to process and analyze the observation surfaces.

#### 2.3.5. SEM Analysis

The specimens were prepared and cured at 20 ± 2 °C with a relative humidity (RH) of 60 ± 5% for 28 days. The specimens were then crushed, and block samples with well-preserved morphology were selected. These samples were immersed in absolute alcohol for 48 h to halt further hydration. Subsequently, the specimens were dried in a vacuum oven at 65 °C for 15 h. The morphology of the dried samples was then examined using scanning electron microscopy (SEM, TESCAN MIRA LMS, Brno, Czech Republic ).

## 3. Results and Discussions

### 3.1. Effects of ADC on the Fluidity and Setting Time of UHPC

**Figure 5:**
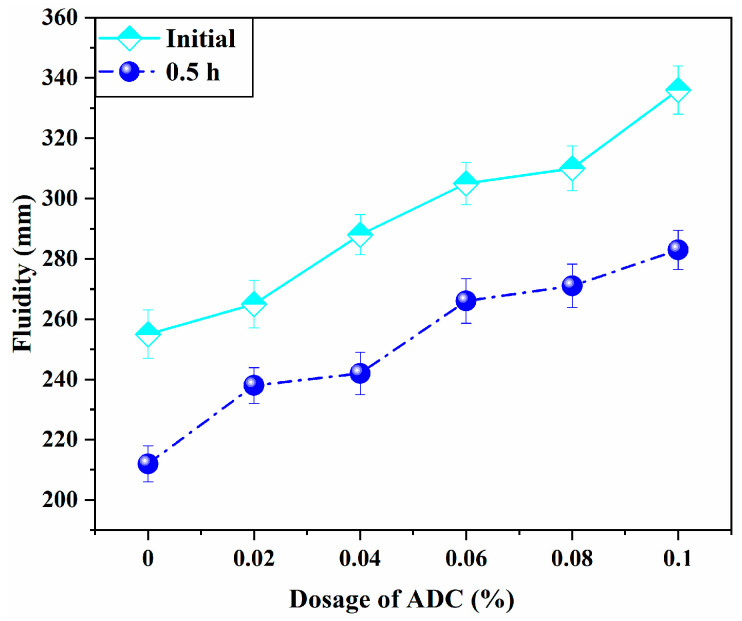
Influence of the addition of ADC on the fluidity of UHPC.

**Figure 6:**
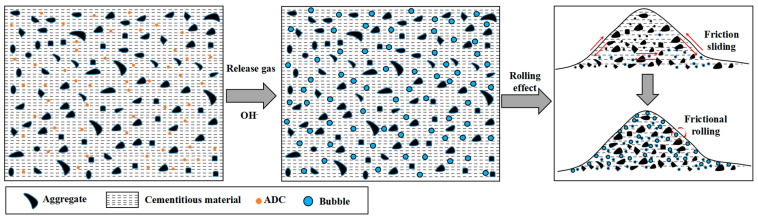
Schematic diagram of the effect of ADC on the fluidity of UHPC.

The fluidity results of UHPC are shown in Figure 5. The truncated cone fluidity of the control group reached up to 250 mm without any bleeding or segregation, indicating excellent workability for the basic mix proportion. As the dosage of ADC increased, the initial fluidity of UHPC exhibited an upward trend. However, when the ADC dosage exceeded 0.06%, no further significant change in fluidity was observed. After 30 min, the fluidity of each experimental group remained higher than that of the control group, following a consistent trend. These observations suggest that ADC enhances the fluidity of UHPC to a certain extent. As discussed in Section 2.1, ADC releases N_2_ and NH_3_ under specific temperature and alkaline conditions during cement hydration, leading to the formation of numerous small, discrete bubbles within the grout. These bubbles act as “rolling balls,” shifting the internal relative motion of the UHPC grout from frictional sliding to frictional rolling. This transformation reduces internal frictional resistance and facilitates the flow of cementitious materials and aggregates, as shown in Figure 6.

**Figure 7:**
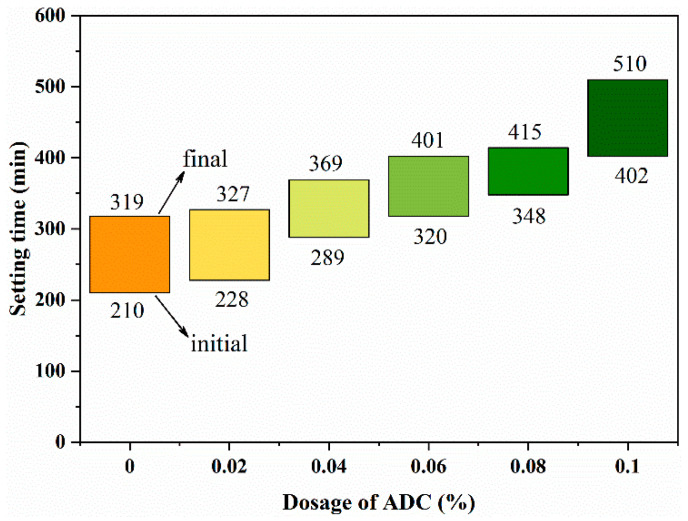
Influence of the addition of ADC on the setting time of UHPC.

The setting time results are shown in Figure 7. It is evident that the addition of ADC significantly influenced the initial and final setting times of UHPC. As the ADC dosage increased, the setting time of UHPC also increased. For instance, the initial setting time of the control sample was 210 min, while samples containing 0.02%, 0.04%, 0.06%, and 0.08% ADC exhibited increases of 9.6%, 37.1%, 51.4%, and 65.7%, respectively. The effect on setting time became more pronounced with higher ADC dosages. Consequently, at an ADC dosage of 0.1%, the initial and final setting times increased by 91.4% and 60.4%, respectively. This phenomenon can be attributed to the presence of abundant bubbles, which reduced the cohesion and yield stress of the UHPC mixtures, thereby extending the setting time.

### 3.2. Effect of ADC on the Mechanical Properties of UHPC

**Figure 8:**
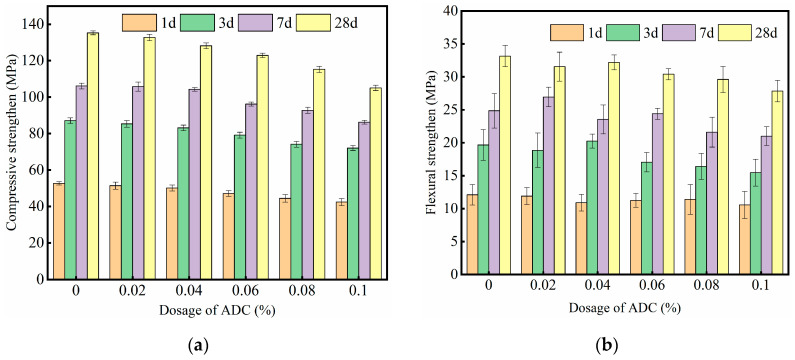
Effect of different dosage of ADC on the mechanical properties of UHPC: (**a**) compressive strength; (**b**) flexural strength.

Figure 8 presents the effect of ADC on the mechanical properties of UHPC. It is evident that the addition of ADC reduced the compressive strength of UHPC to varying degrees. When the ADC dosage was 0.02% and 0.04%, the reduction in compressive strength was relatively minor, with a decrease of less than 5% across different ages compared to the control group. However, as the ADC dosage increased, the strength reduction became more significant. In particular, at an ADC dosage of 0.1%, the compressive strength of UHPC decreased by 17.8%, 18.9%, 20.2%, and 21.1% at 1, 3, 7, and 28 days, respectively, indicating a more substantial impact on later-stage strength.

The flexural strength results, shown in Figure 8b, exhibited a similar trend. The flexural strength of UHPC decreased as the ADC dosage increased. When the ADC dosage was below 0.06%, the reduction in flexural strength was less than 5% compared to the control group. However, when the dosage exceeded 0.06%, the flexural strength decreased by more than 10% at different ages, with a more pronounced effect at later stages. ADC is a chemical additive that primarily exerts an expansive effect during the plastic stage of cement-based materials by releasing gas. These gases reduce the compactness of the material and alter the pore structure, resulting in a decrease in overall strength. The experimental results confirm that the incorporation of ADC leads to a reduction in UHPC strength.

### 3.3. Effect of ADC on the Vertical Expansion Rate of UHPC

**Figure 9:**
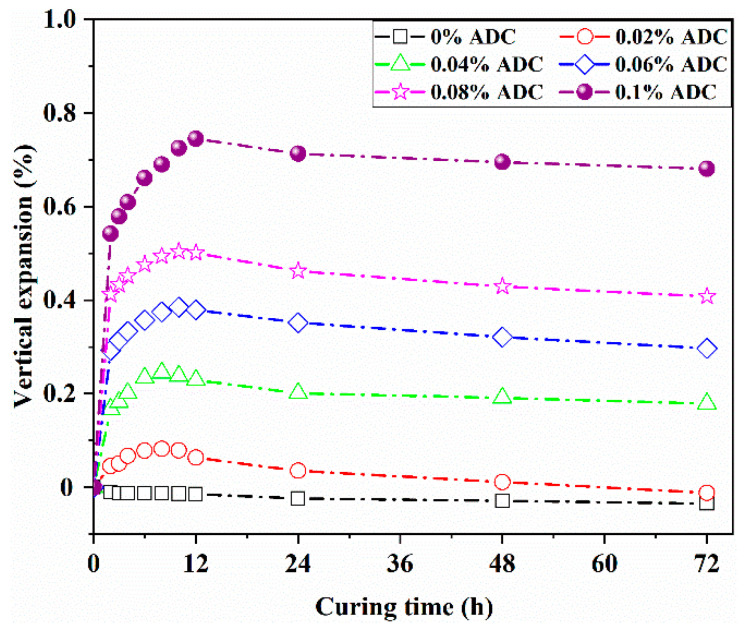
Vertical expansion rate of UHPC with different ADC dosages.

The early strength of UHPC develops rapidly, making the control of early shrinkage critically important. This study analyzed the effect of ADC on the vertical expansion rate of UHPC at 2, 3, 4, 6, 8, 10, 12, 24, 48, and 72 h, with the results presented in Figure 9. It was observed that the control group began to contract from 2 h, with this contraction progressively increasing as the concrete aged.

**Figure 10:**
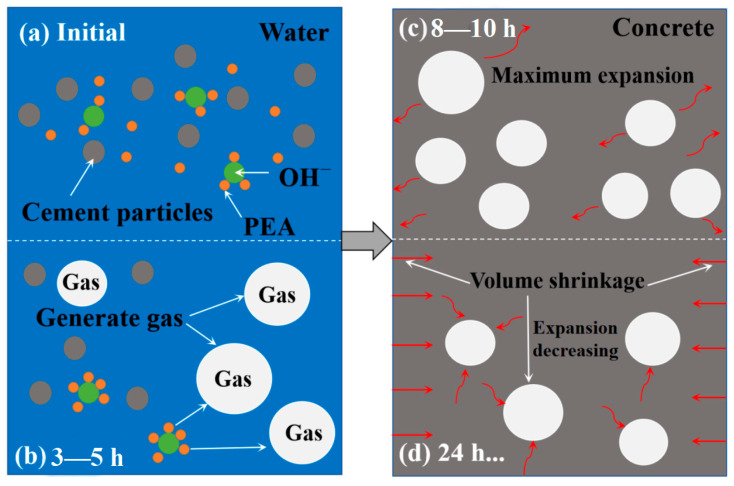
Schematic illustration of the plastic expansion effect of ADC over time.

For the UHPC group containing 0.02% ADC, the highest vertical expansion rate was observed at 8 h, but by 24 h, the expansion rate turned negative compared to the 3 h rate, indicating shrinkage. In contrast, when the ADC dosage ranged from 0.04% to 0.1%, the UHPC remained in an expansion state within the first 24 h. The differences in expansion rates between 24 and 3 h for these groups were 0.019%, 0.038%, 0.029%, and 0.134%, respectively. Notably, the maximum vertical expansion rate was reached at 10 h for groups with 0.04%, 0.06%, and 0.08% ADC, while the group with 0.1% ADC reached its peak at 12 h. Additionally, slight shrinkage was observed in all groups at 48 and 72 h compared to their expansion at 24 h. Figure 10 provides a schematic diagram illustrating the plastic expansion effect of ADC over time. The expansion effect of ADC primarily occurs during the plastic stage of UHPC, with gas generation peaking around 3–5 h and then slowing significantly between 5 and 24 h. For UHPC with a low water-to-binder ratio and a high cementitious material content, the volume shrinkage eventually surpasses the expansion induced by ADC after 24 h, leading to a rebound effect in vertical expansion.

### 3.4. Effect of ADC on the Pore Structure of UHPC

**Figure 11:**
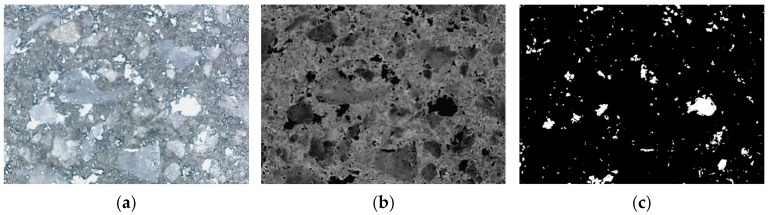
The pore structure of UHPC specimens with the ADC dosage of 0.06%: (**a**) original image, (**b**) gray processing, (**c**) binarization processing.

**Figure 12:**
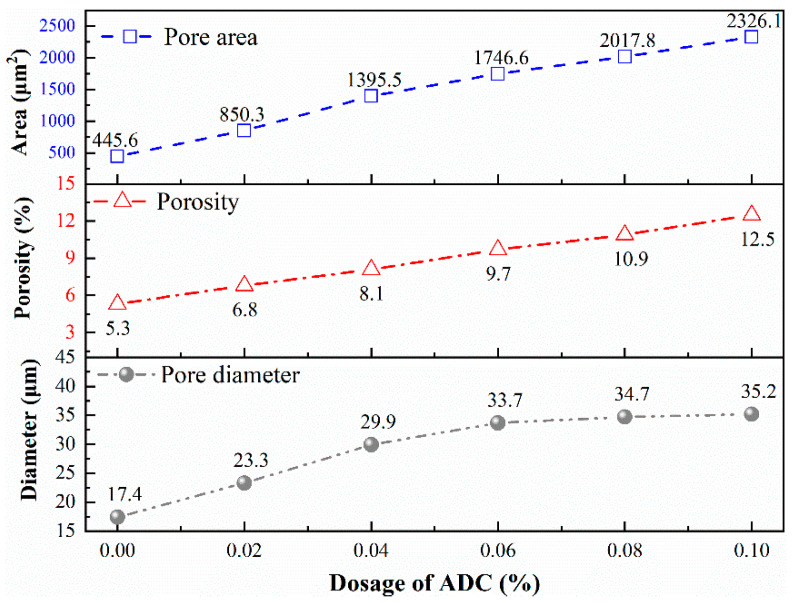
The effect of ADC on the pore structure parameters of UHPC.

To investigate the effect of ADC on the internal pore structure of UHPC, a digital microscope was employed to capture the cross-sectional images of the test specimens. The captured images were processed using Image-Pro Plus software, where they were converted to grayscale and then binarized. Figure 11 illustrates the pore structure of UHPC specimens containing 0.06% ADC. The pore structure parameters of UHPC with varying ADC dosages were measured and statistically analyzed at 1 day, focusing on parameters such as porosity, pore area, and average pore size. The results are presented in Figure 12.

The analysis revealed that the average pore area, porosity, and pore diameter increased with rising ADC dosage. Compared to the control group, the porosity in the experimental groups increased by 28%, 52%, 83%, 105%, and 136%, respectively, as the ADC dosage increased. During the plastic stage of UHPC, ADC continuously releases gas, which becomes distributed throughout the slurry. As the hydration process progresses, the cement slurry begins to solidify, gaining strength and trapping some bubble within the UHPC, ultimately forming numerous micropores in the solidified structure. The increase in pore parameters leads to a reduction in the compactness of UHPC. Consequently, while the addition of ADC effectively controls shrinkage during the plastic stage, it also results in a decrease in the compressive strength of UHPC as the ADC dosage increases.

### 3.5. Effect of ADC on the Microstructure of UHPC

**Figure 13:**
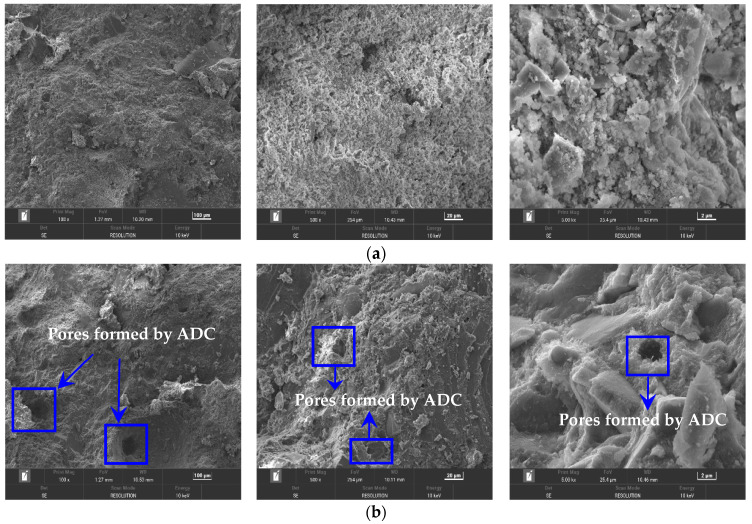
SEM image of UHPC at 28 d: (**a**) control group and (**b**) experimental group with 0.06% ADC.

ADC generates a substantial amount of gas during the hydration process of UHPC, which enhances fluidity, prolongs setting time, and effectively addresses shrinkage during the plastic stage. However, this also results in a reduction in compressive strength. Based on these experimental findings, it is essential to study the impact of ADC on the microstructure of UHPC. Figure 13 presents the microstructure of both the control group and the experimental group with 0.06% ADC at 28 days. The control group exhibited fewer pores and maintained a relatively dense internal structure. In contrast, the UHPC group containing 0.06% ADC displayed the presence of pores, which reduced the overall compactness and consequently led to a decrease in compressive strength. Both the control and experimental groups formed a certain amount of hydration products, including C-S-H gel, calcium hydroxide, and ettringite, contributing to the compact microstructure of UHPC. However, the density of these hydrates was lower in the experimental group with 0.06% ADC compared to the control group. As the ADC dosage increased, the compressive strength of the specimens decreased significantly, which can be attributed to the combined effects of ADC on the hydration process and microstructure of UHPC.

## 4. Conclusions

This study experimentally investigated the effects of ADC on the flowability, setting time, mechanical properties, and plastic shrinkage of UHPC, along with an analysis of the pore structure parameters and microstructure. Based on the experimental findings, the following conclusions can be drawn:

(1) Impact on Flowability and Setting Time: ADC releases gas under the alkaline conditions of cement hydration, which reduces internal frictional resistance and enhances the fluidity of UHPC by facilitating the movement of cementitious materials and aggregates. The addition of ADC also significantly influences both the initial and final setting times, with prolonged setting times observed as the ADC dosage increases.

(2) Effect on Mechanical Properties: The gas-releasing expansion effect of ADC leads to a reduction in the compactness of UHPC and alters its pore structure. Consequently, the incorporation of ADC decreases the compressive and flexural strength of UHPC to varying extents, particularly when the ADC dosage exceeds 0.04%. At higher dosages, the strength reduction becomes more pronounced, indicating a trade-off between shrinkage control and mechanical strength.

(3) Control of Plastic Shrinkage: ADC effectively addresses early shrinkage in UHPC by exerting its expansion effect predominantly during the plastic stage. The experimental results showed that the maximum vertical expansion rate occurred between 8 and 12 h, with UHPC remaining in an expansion state within 24 h at ADC dosages ranging from 0.04% to 0.1%. The differences in expansion rates between 24 and 3 h were 0.019%, 0.038%, 0.029%, and 0.134%, respectively.

(4) Influence on Pore Structure: The continuous gas release from ADC during the plastic stage results in the formation of numerous micropores within the solidified UHPC matrix. While this effectively mitigates plastic shrinkage, it also increases pore structure parameters such as porosity and pore area, thereby reducing the overall compactness of UHPC. This increased porosity contributes to the observed decrease in mechanical strength, highlighting the need for careful optimization of ADC dosage to balance shrinkage control with strength requirements. From the experimental results, it appears that the ADC dosage of 0.04–0.06% is appropriate, as it can effectively control the plastic shrinkage of UHPC without significantly affecting its mechanical strength.

## Figures and Tables

**Table 1:** Chemical compositions of cementitious materials and ADC.

Materials	Chemical Composition (%)							
	SiO_2_	Al_2_O_3_	CaO	Fe_2_O_3_	SO_3_	MgO	K_2_O	Na_2_O
OPC	20.91	4.95	63.28	3.45	3.11	1.01	0.94	0.78
MP	34.21	14.15	39.11	0.82	0.12	0.32	1.95	0.58
SF	91.95	1.15	0.63	0.85	0.22	1.08	1.68	0.45
FA	60.86	21.7	3.48	5.48	0.31	1.75	1.67	0.83

**Table 2:** Mechanical and geometric properties of employed fibers.

Fiber Type	Length (mm)	Diameter (mm)	Tensile Strength (MPa)	Elastic Modulus (GPa)
CSF	13	0.2	2800	205

**Table 3:** Mixture proportions of UHPC.

Mixture	Materials Dosage/g								
	OPC	MP	SF	FA	Sand	ADC	SP	CSF	W/B
ADC-0	600	150	140	60	1000	0	6	150	0.18
ADC-0.02%	600	150	140	60	1000	0.2	6	150	0.18
ADC-0.04%	600	150	140	60	1000	0.4	6	150	0.18
ADC-0.06%	600	150	140	60	1000	0.6	6	150	0.18
ADC-0.08%	600	150	140	60	1000	0.8	6	150	0.18
ADC-0.10%	600	150	140	60	1000	1	6	150	0.18

## Data Availability

The original contributions presented in this study are included in the article. Further inquiries can be directed to the corresponding author.
